# Therapeutic Potential of Targeting *USP2* in Pediatric Group 3 and 4 Medulloblastomas: Insights from In Silico, Ex Vivo, and In Vitro Studies

**DOI:** 10.1007/s12311-026-02051-w

**Published:** 2026-07-09

**Authors:** Jonas José da Silva, Manuela Eduarda de França, Alcides Euzebio Tavares Xavier, Mariane Minussi Baptistella, Eduardo Afonso da Silva Pereira, Marina Ferreira Cândido, Luis Fernando Peinado Nagano, Jéssica Oliveira de Santis, Rosane Gomes de Paula Queiroz, Silvia Regina Brandalise, José Andres Yunes, Luiz Gonzaga Tone, Elvis Terci Valera, Pablo Shimaoka Chagas, Carlos Alberto Scrideli

**Affiliations:** 1 ¹Department of Genetics, Ribeirão Preto Medical School, University of São Paulo, Ribeirão Preto, São Paulo, Brazil; 2 Department of Pediatrics, Ribeirão Preto Medical School, University of São Paulo, Av. Bandeirantes, 3900, Ribeirão Preto, São Paulo 14048- 900 Brazil; 3 Boldrini Children’s Center, Campinas, Brazil; 4 Department of Clinical Analysis, Toxicology, and Food Science, School of Pharmaceutical Sciences of Ribeirão Preto, University of São Paulo, São Paulo, Brazil; 5 National Science and Technology Institute for Children’s Cancer Biology and Pediatric Oncology – INCT BioOncoPed, Porto Alegre, Brazil

**Keywords:** Medulloblastoma, USP2, Group 3/4, Therapeutics

## Abstract

Medulloblastoma (MB), the most common malignant pediatric brain tumor, is classified into four molecular groups: WNT, SHH, G3, and G4. G3 and G4 MBs are characterized by poor prognosis and high metastatic potential. Although USP2 (ubiquitin-specific peptidase 2) has been described as a prognostic biomarker and a promising therapeutic target in several cancer types, its role in the pathogenesis of G3 and G4 MBs remains unexplored. In this study, USP2 expression was investigated through in silico analyses, public datasets (GSE85217), and tumor samples from 54 pediatric MB patients. In addition, the effects of USP2 inhibition were evaluated in two G3/G4 MB cell lines (D283 Med and USP-13) using ML364, a selective USP2 inhibitor. USP2 was overexpressed in G3 and G4 MBs and was associated with metastasis and unfavorable clinical outcomes. Furthermore, ML364 treatment reduced USP2 protein expression level, as well as cell viability, migration, invasion, and colony-forming capacity in both cell lines. These findings indicate that USP2 is upregulated in pediatric G3 and G4 MBs and support its potential role as a biomarker of poor prognosis and a therapeutic target in these molecular subgroups.

**Supplementary Information:**

The online version contains supplementary material available at 10.1007/s12311-026-02051-w.

## Introduction

Medulloblastoma (MB), a malignant embryonal tumor originating from cerebellar granule progenitor cells [1], is classified as grade IV by the World Health Organization. Although MB can occur in adults, its incidence peaks at ages 1 to 4 and 5 to 9 years, and it affects mainly males [1, 2]. On the basis of molecular profile, MB is categorized into four groups: WNT (Wingless), SHH (Sonic Hedgehog), Group 3 (G3), or Group 4 (G4) [3]. Together with clinical practice, this categorization has improved the stratification of patients aiming at better therapeutic direction. WNT MB has the best prognosis and represents about 10% of MB cases. SHH MB represents about 30% of MB diagnoses, and the *TP53* mutational status has been associated with distinct outcomes in this group—tumors with *TP53-*wildtype have a favorable prognosis, while *TP53* mutations are associated with a dismal prognosis [3–5]. G3 MB, representing 25% of MB cases, is highly metastatic and has the worst prognosis. G4 MB represents 35% of MB cases, has the highest incidence at diagnosis, and presents intermediate prognosis [3, 5]. Molecular classification of G3 and G4 MBs may be inconclusive because these groups exhibit strong molecular overlap. Furthermore, in contrast to WNT and SHH MBs, a signaling pathway has not been characterized or associated with the onset and progression of G3 and G4 MBs. Thus, no potential targeted therapies exist to treat G3 and G4 MBs alongside current treatment protocols [2, 5].

In this scenario, identifying new targets (biomarkers) with potential therapeutic purposes for G3 and G4 MBs is paramount. Our research group has conducted thorough RNA sequencing analysis (RNAseq – GSE181293 database) and identified differentially expressed genes (DEGs) in G3 MB [6]. This analysis helped us to identify the Ubiquitin Specific Peptidase 2 (*USP2*) gene, a promising therapeutic target in several human tumors [7, 8], as a biomarker of poor prognosis. The *USP2* gene is located on the long arm of chromosome 11, band 23.3 (11q23.3), and encodes USP2, a protein with ubiquitin protease function, that is, a deubiquitylating enzyme (DUB). USP2 acts as an oncogenic protein by deubiquitylating and stabilizing other oncogenic proteins, such as MDM2, Aurora-A, and TGF-B, blocking apoptosis, accelerating the cell cycle, and promoting metastasis [9]. More recently, USP2 has been described to deubiquitylate the protein SKP2 and hence stabilize its expression [10]. This information is important because SKP2 is involved in many biological processes, particularly the cell cycle, and participates in the degradation of tumor suppressors, specifically p27 and p21 [9]. SKP2 overexpression and reduced or absent p27 expression have been associated with progression and poor prognosis of several malignant tumors [10, 11].

In the context of tumors, *USP2* contributes to metastatic lesions of triple-negative breast cancer and to chemotherapy resistance in prostate and lung cancer cells. Moreover, overexpressed *USP2* has been correlated with increased tumor cell proliferation, migration, and invasion in ovarian cancer and hepatocellular carcinoma [7, 12–16]. Given that altered *USP2* expression in the cellular microenvironment can cause aggressive tumor phenotypes to emerge, it is essential to understand the role played by this gene in stimulating tumor cell proliferation, migration, and invasion in G3 and G4 MBs. Therefore, this study has investigated the biological and molecular functions of *USP2* by assessing its expression in a Brazilian cohort and by using MB cell lines to conduct representative functional assays.

## Subjects and Methods

### Patient Samples

This study included 54 MB samples obtained from patients aged 0 to 18 years assisted at the University Hospital of the Ribeirão Preto Medical School – University of São Paulo (FMRP) or the Boldrini Children’s Center (CIB). The samples were molecularly characterized as SHH MB (*n* = 13), WNT MB (*n* = 14), G3 MB (*n* = 9), or G4 MB (*n* = 18), as described in [17]. Immediately after surgery, the tumor samples were frozen at -80 °C and stored. All the samples were collected after the patients or their legal guardians signed the informed consent form. This project was approved by the local Research Ethics Committee (CAAE no. 68159623.0.0000.5440).

### RNA Extraction, cDNA Synthesis, and qRT-PCR

Total RNA in the biological samples obtained from the patients was extracted by using the TRIZOL^®^ reagent (Invitrogen Inc, Carlsbad, CA, USA). cDNA was synthesized by employing the High Capacity^®^ kit (Applied Biosystems, Foster City, CA, USA) and following the manufacturer’s protocol. *USP2* expression was analyzed by using the real-time quantitative PCR (RT-qPCR) technique. All the reactions were performed on the Quant Studio 12 K apparatus (Applied Biosystems, Foster City, CA, USA). The *HPRT* gene was employed as endogenous control. The mean gene expression of six non-neoplastic cerebellar samples was used as calibrator. qRT-PCR was carried out by employing primers designed for the specific regions of the genes of interest (Supplementary Table 1). The relative quantification of gene expression was determined by using the 2-ΔΔCT method [18].

### Cell Lines and Culture Conditions

*USP2* expression was analyzed in the human MB cell lines D283 Med and USP-13 (G3/4); CHLA (G4); and ONS-76, UW228, UW402, DAOY, and UW473 (SHH). The G3/4 MB cell lines D283 Med and USP-13 were chosen for functional studies. The *MYCC* and *OTX2* genes are amplified in D283 Med cells. In USP-13 cells, *OTX2* is amplified, but *MYCC* is not (Supplementary Table 2). The cell lines were cultured in Dulbecco’s Modified Eagle Medium/DMEM (Sigma-Aldrich^®^ #D5030) supplemented with 10% fetal bovine serum (FBS) (Vitrocell #S0011), 100 mg/mL streptomycin (Sigma-Aldrich^®^ #3810-74-0), and 100 U/mL penicillin (Sigma-Aldrich^®^ #69-57-8). The cultures were carried out in humidified atmosphere containing 5% CO_2_ at 37 °C. The cell lines were authenticated by using STR markers and validated by verifying the ATCC profile. All the cell lines tested negative for mycoplasma.

### Validation of *USP2* Expression in Public Databases

The R2: Genomics Analysis and Visualization platform (https://r2.amc.nl/) was used to evaluate *USP2* expression data in the MB samples and MB molecular groups available in public databases. This platform allows the expression of several genes to be evaluated in different databases. For the comparative analysis of *USP2* expression in the MB groups, the GSE85217 database (*n* = 628 patients) was employed [3].

### Cell Preparation and Treatment with ML364

For the cell viability assays, D283 Med or USP-13 cells were treated with different ML364 doses ranging from 5 to 150 µM for 24, 48, or 72 h. ML364 (Cat. No.: HY-100900; MedChemExpress), which selectively inhibits the USP2 protein, had previously been diluted in DMSO, according to the manufacturer’s recommendation.

### Western Blot

Proteins were extracted from the cell cultures by using RIPA Lysis and Extraction Buffer^®^ (Pierce Biotechnology, IL, USA), according to the manufacturer’s instructions. The proteins were quantified by employing the Protein Assay Dye Reagent Concentrate (Bradford, Bio-Rad Laboratories, Hercules, CA, USA). To analyze the proteins, 40–50 µg of each sample was separated by SDS-PAGE electrophoresis. The proteins were transferred to nitrocellulose membranes and incubated in 1% TBST containing 5% blocking solution at room temperature for 1 h. Then, the membranes were incubated with the primary antibody for the protein of interest, diluted according to the manufacturer’s instructions at 4 °C overnight (Supplementary Table 3). Next, the membranes were washed with 1% TBST and incubated with the secondary antibody at room temperature for 1 h. The secondary antibody was visualized by using the ECL™ Western Blotting Analysis System (Amersham GE Healthcare, Buckinghamshire, UK). Band intensity was analyzed to quantify protein expression by employing the ImageJ 1.52a software (National Institutes of Health, Bethesda, Maryland, USA). The experiments were conducted in triplicate.

### Functional Assays

#### Clonogenic Assay

On the basis of cell size and doubling time, 300 USP-13 or 500 D283 Med cells/well were plated in six-well plates and incubated at 37 °C for 14 days. After incubation, the plates were washed with PBS, fixed in methanol for 20 min, and stained with Giemsa and phosphate buffer (1:15) for 30–40 min, which was followed by a water wash sequence. Finally, the cells were counted by using a stereomicroscope (40x magnification) [19]. The experiments were performed in independent biological and experimental triplicates.

#### Cell Viability Assay

Cell viability was assayed in 96-well plates. To this end, 2 × 10³ USP-13 or 5 × 10³ D283 Med cells/well were seeded. Then, the cells were incubated under 5% CO₂ at 37 °C for 24, 48, or 72 h. After each time interval, 10% volume of MTT (3-(4,5-dimethylthiazol-2-yl)-2,5-diphenyltetrazolium bromide) was added, and the plates were incubated for additional 4 h. Absorbance at 570 nm was read on the iMark Microplate Absorbance Reader (BioRad Laboratories). The experiments were accomplished in independent biological and experimental triplicates.

### Transwell Assays

#### Transwell Migration Experiment

For the Transwell experiments, 24-well ThinCert^®^ plates (Greiner Bio-One, Kremsmünster, Austria) were used. A total of 1 × 10⁵ D283 Med or USP-13 cells (control or treated with ML364 at the *IC*_*50*_ concentration) were seeded in 400 µL of serum-free minimal medium in the upper chamber of the 24-well plates. The plates were gently shaken so that the cells were better distributed in the chamber. In the lower compartment of the insert, 500 µL of complete culture medium containing 10% fetal bovine serum was added to stimulate migration. Then, the cells were incubated under 5% CO₂ at 37 °C for 48 h. The experiments were performed in independent biological and experimental triplicates.

#### Transwell Onvasion Experiment

The insert was coated with 100 µL of Geltrex™ hESC-Qualifield (Gibco, NY, USA), which was followed by drying at room temperature for 1 h to create a membrane on the bottom of the insert. Then, 1 × 10^5^ D283 Med or USP-13 cells (control or treated with ML364 at the *IC*_*50*_ concentration) were seeded in 400 µL of serum-free minimal medium in the upper chamber of the 24-well plates. Then, 500 µL of complete medium containing 10% serum was added to the lower chamber of the 24-well plates. The cells were incubated under 5% CO₂ at 37 °C for 48 h. After incubation, a cotton swab was used to wipe the top of the insert to remove any cells that had not invaded the bottom. Methanol was added to the bottom of the insert to fix the cells for 20 min. Next, the cells were stained with Giemsa for 20 min. After the washing steps and once the insert was dry, images were acquired at 4x magnification under an inverted microscope. The experiments were conducted in independent biological and experimental triplicates.

#### Analysis of Results

Statistical analysis was performed by using SPSS 20.0 for Windows (SPSS, Chicago, IL, USA) and GraphPad Prism 8.0 (GraphPad Software, San Diego, CA, USA). To analyze gene expression comparisons between the different samples and the clinical and biological characteristics, the Mann-Whitney and Kruskal-Wallis tests (with Dunn’s post-test) were employed. To calculate the overall survival (considered from the date of diagnosis to the date of death or last follow-up), Kaplan-Meier curves and the Log-Rank test were used. For the functional assays of cell viability, clonogenicity, migration, and invasion, the Student t-test, two-way ANOVA, and the Bonferroni post-test were employed. The ROC curve test was used to evaluate the potential of *USP2* to discriminate between MB molecular groups. The experiments were carried out in independent biological and experimental triplicates. The results were considered statistically significant when *p* ≤ 0.05.

## Results

### *USP2* Expression was Higher in G3 and G4 Pediatric MB Samples

**Fig. 1:**
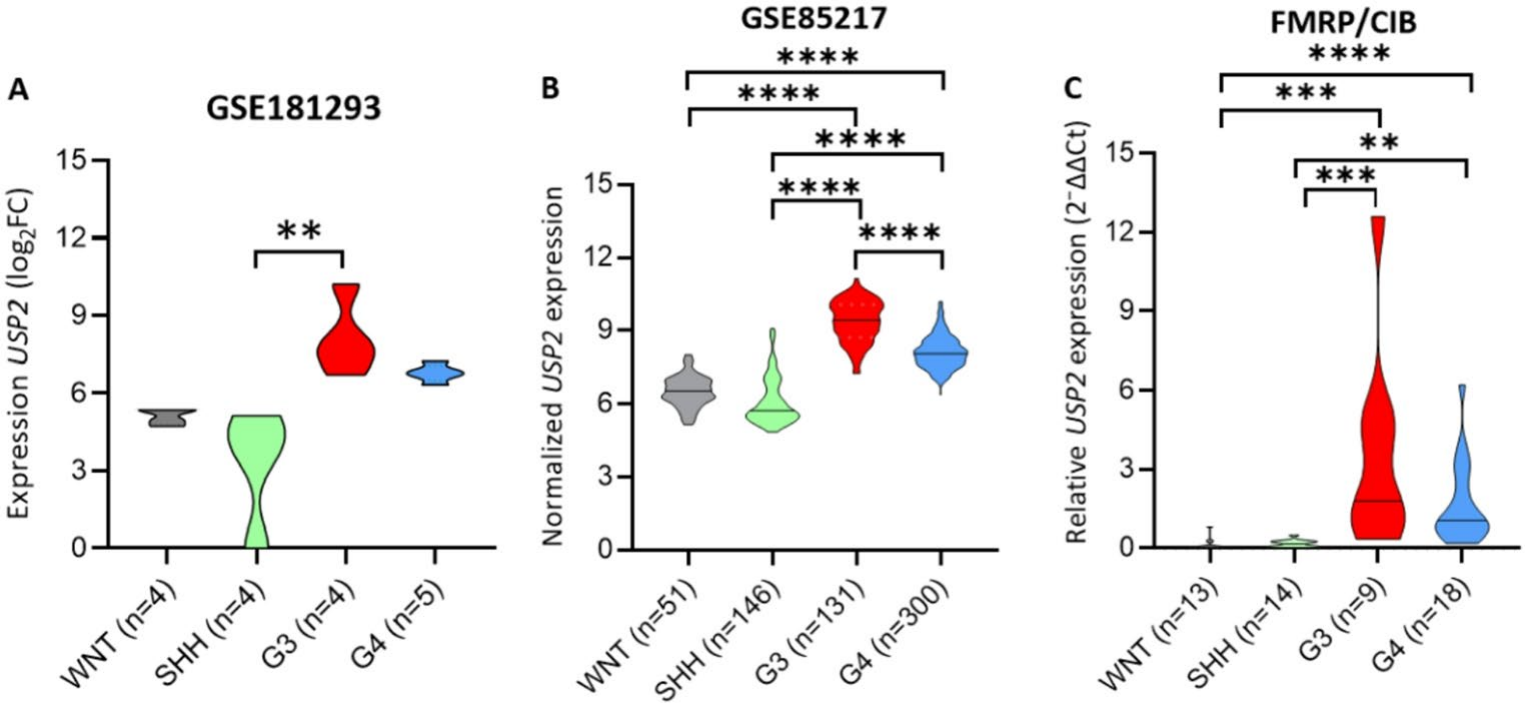
USP2 gene expression in pediatric medulloblastoma (MB) samples. **A** *USP2* gene expression levels (log2FC) in our RNA-seq dataset GSE181293 (*n* = 17). Statistical comparisons were performed among all molecular subgroups using the Kruskal–Wallis test followed by Dunn’s multiple-comparison test; only statistically significant comparisons are displayed. G3 tumors showed significantly higher USP2 expression than the SHH subgroup. **B** *USP2* overexpression in G3 and G4 MBs compared to WNT and SHH MBs in the GSE85217 cohort (*n* = 628). **C** *USP2* overexpression in G3 and G4 MBs compared to WNT and SHH MBs in the FMRP/CIB cohort (*n* = 54). Kruskal–Wallis test with Dunn’s multiple-comparison post-test. **(*p* = 0.0017), **(*p* < 0.0060), ***(*p* < 0.0004), ****(*p* < 0.0001)

First, we used RNAseq data, (n = 17) from our research group, (GSE181293) [6], (Fig. 1A) and validated in the GSE85217 dataset, (n = 628) [3] to evaluate differential *USP2* expression in pediatric MBs, (Fig. 1B). Next, we evaluated *USP2* expression in the FMRP/CIB cohort of pediatric MB, (n = 54), (Fig. 1C). Supplementary Table 4 lists the clinical data of these patients. In the GSE85217 and FMRP/CIB cohorts, *USP2* expression was significantly higher in G3 and G4 MBs compared to the WNT and SHH molecular groups. In our RNA-seq dataset, a similar trend was observed, with G3 tumors showing significantly higher *USP2* expression than the SHH subgroup.

### *USP2 *Overexpression was Associated with Metastatic Disease in Pediatric MB Patients

**Fig. 2:**
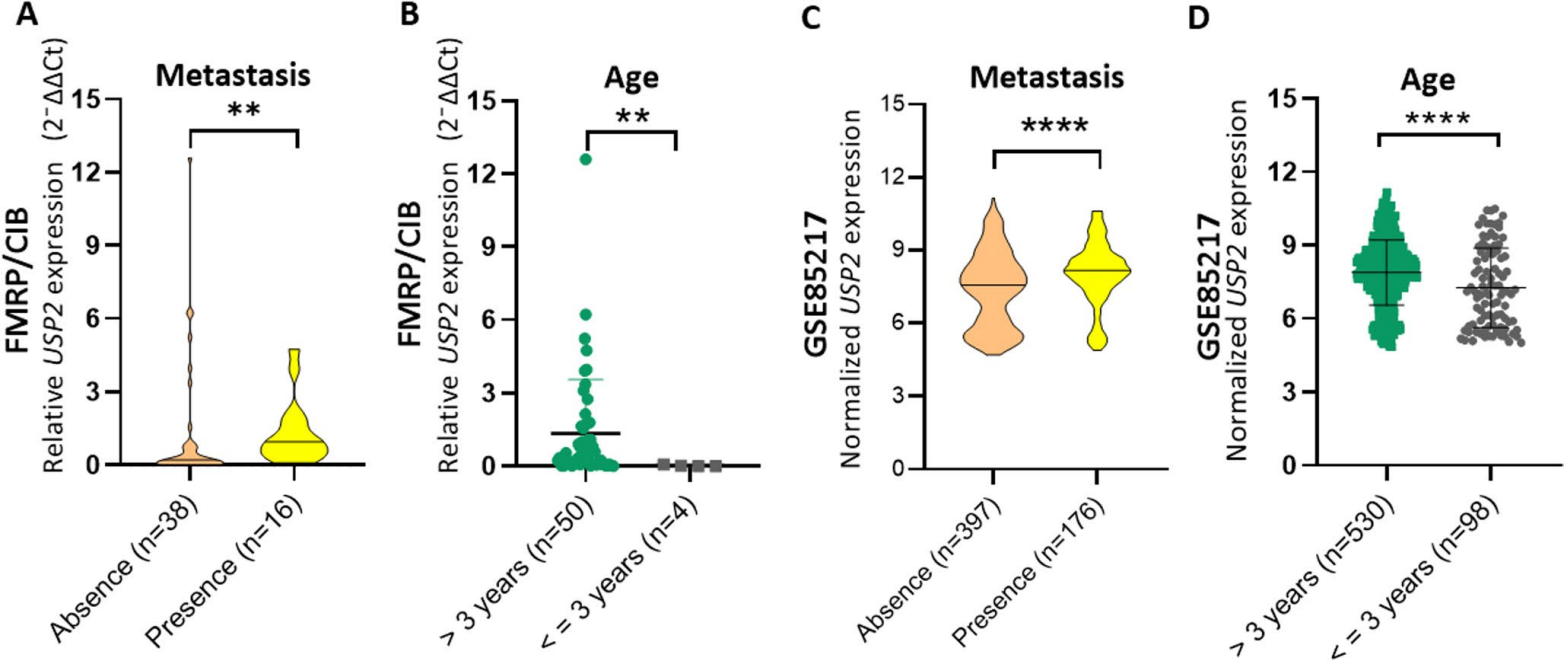
Association of *USP2 *expression with clinical-pathological features in pediatric medulloblastoma (MB).** A ***USP2* expression according to the presence (*n* = 16) or absence (*n* = 38) of metastasis in the FMRP/CIB cohort. **B ***USP2* expression stratified by patient age (> 3 years, *n* = 50 vs. ≤ 3 years, *n* = 4) in the FMRP/CIB cohort. **C ***USP2* expression according to the presence (*n* = 176) or absence (*n* = 397) of metastasis in the GSE85217 database. **D ***USP2* expression stratified by patient age (> 3 years, *n* = 530 vs. ≤ 3 years, *n* = 98) in the GSE85217 database. Data analyzed using the Mann-Whitney test **(*p* < 0.001) ****(*p* < 0.0001). Note: In the GSE85217 metastatic profile analysis, 130 patients with missing clinical data were excluded

Analysis of the clinical characteristics of our cohort of 54 pediatric MB patients showed higher *USP2* expression in patients with metastatic disease (*p* = 0.001) and aged over three years (*p* = 0.001) (Fig. 2A and B). The other analyzed parameters (gender, degree of surgical resection, and recurrence) did not reveal statistical significance (data not shown). Analysis of these characteristics in the GSE85217 database (*n* = 628) helped to validate the results—*USP2* expression was higher in patients with metastatic disease (*P* < 0.0001) and aged over three years (*P* < 0.0001) (Fig. 2C and D).

### Analyzing whether *USP2*has Potential Prognostic Value in G3 and G4 MBs

**Fig. 3:**
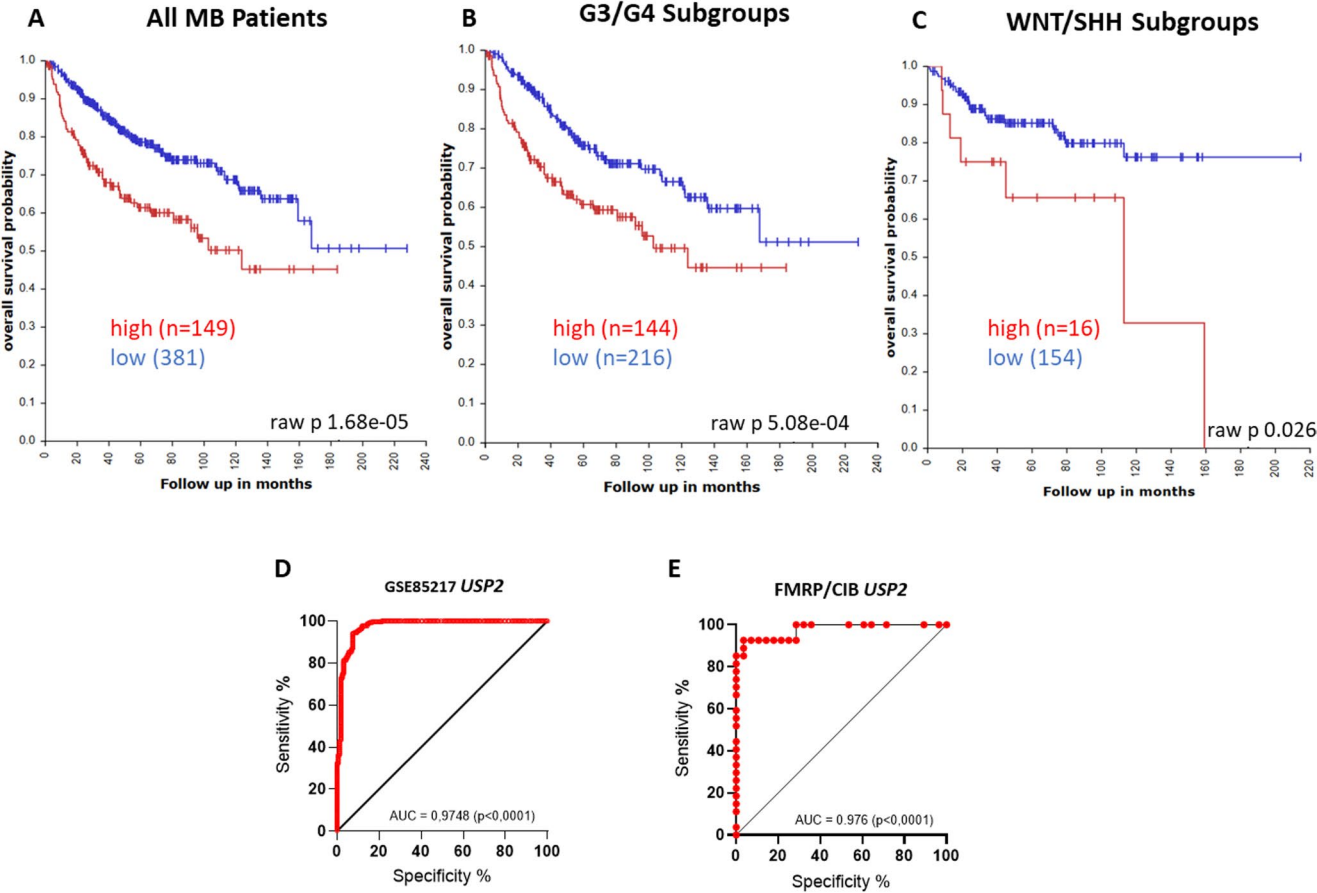
Overall survival and ROC curve analysis for MB patients across the GSE85217 database and the FMRP/CIB cohort.** A–C** Kaplan-Meier curve and log-rank test showing MB samples in general (*n* = 530), MB samples classified only as G3 and G4 (*n* = 360) and MB sample classified WNT and SHH (*n* = 170), respectively, showing that high *USP2* expression (red) is associated with lower survival among patients compared to tumors with low *USP2* expression (blue). **D** and **E** Diagnostic value of *USP2* in discriminating against the MB molecular groups WNT/SHH and G3/G4 based on *USP2* expression levels. GSE85217 (*n* = 628), FMRP/CIB (*n* = 54) by ROC curve analysis. Note: In the analysis of the overall survival in the GSE85217 database, 98 patients did not present this parameter for evaluation and were excluded from the analysis

To assess whether *USP2* overexpression is associated with prognosis, we analyzed the overall survival of patients diagnosed with MB (Fig. 3A) and the overall survival of patients with G3 or G4 MB and WNT or SHH (Fig. 3B, C) by using the public GSE85217 database [3]. *USP2* overexpression was associated with worse overall survival in MB patients regardless of the molecular group (*p* = 0.0000168), being more pronounced in groups G3 and G4 (*p* = 0.000508) compared to groups WNT/SHH. (*p* = 0.026). The sample size of the FMRP/CIB cohort (*n* = 54) was relatively small, and the patients were submitted to one of three treatment protocols, which provided distinct responses. Therefore, it was difficult to analyze overall survival in this cohort of patients adequately.

ROC (receiver operating characteristics) curve analysis showed that *USP2* holds potential for discriminating between WNT/SHH MBs and G3/G4 MBs with a AUC (area under the curve) of 0.9748 (Fig. 3C). Furthermore, applying the same test together with the *USP2* expression data obtained for the FMRP/CIB cohort corroborated the in silico analyses and confirmed that *USP2* can discriminate between MB molecular groups. The AUC for this test was 0.9762 (Fig. 3D), suggesting that *USP2* has potential value for discriminating between G3/G4 and WNT/SHH MBs.

### Analyzing *USP2*and USP2 Expression in D283 Med and USP-13 Cells

**Fig. 4:**
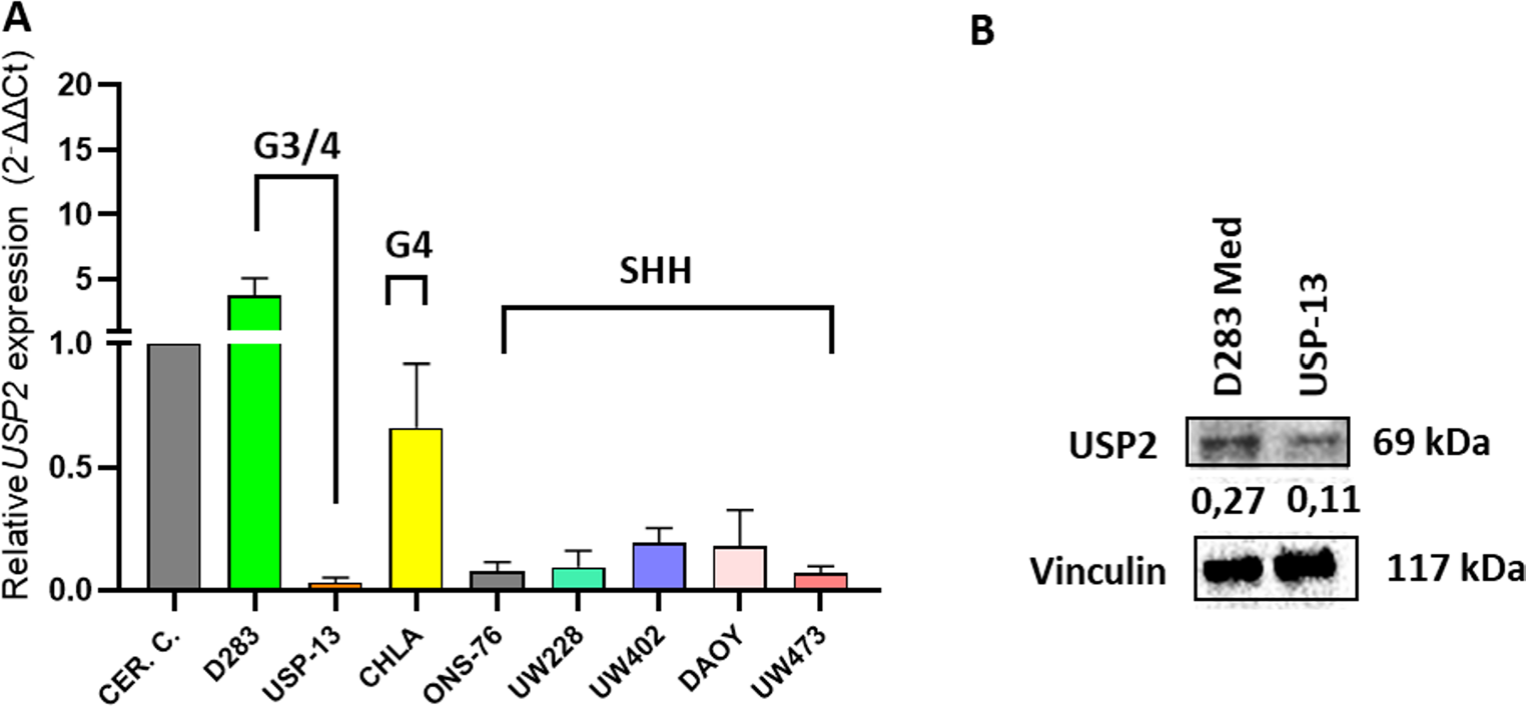
*USP2 *and USP2 expression in the pediatric medulloblastoma (MB) cell lines D283 Med and USP-13. **A** Higher *USP2* expression in D283 Med cells and lower *USP2* expression in other MB cell lines compared to the non-neoplastic cerebellum controls (CER. C.). **B** Higher and lower USP2 expression in D283 Med and USP-13 cells, respectively, compared to endogenous Vinculin

Among the MB cell lines analyzed herein, only D283 Med cells (amplified *MYCC*) showed higher *USP2* expression compared to the non-neoplastic cerebellar control samples (Fig. 4A). Investigating USP2 expression in the pediatric G3/G4 MB cell lines D283 Med and USP-13 helped to extend the analyses by providing two distinct in vitro models with contrasting baseline expression levels. USP2 protein expression was higher in D283 than in USP-13 cells (Fig. 4B). This differential expression pattern reflects the well-known molecular heterogeneity of G3/G4 MBs. Thus, we conducted functional assays in these complementary cell lines in vitro to investigate the functional role played by USP2 in G3 and G4 MBs.

### Cell Viability of D283 Med and USP-13 Cells and USP2 Expression in D283 Med Cells Treated with ML364

**Fig. 5:**
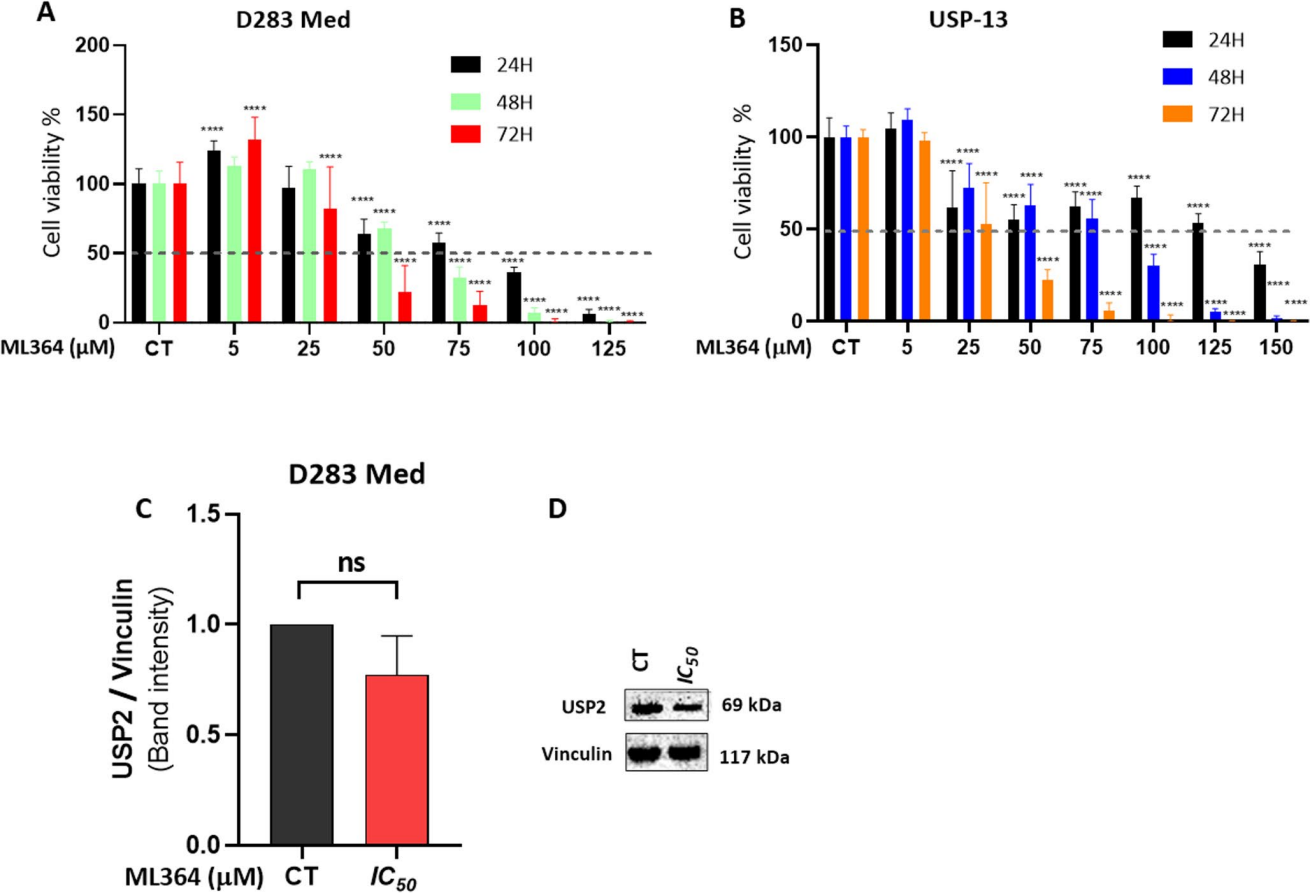
Cell viability analysis and USP2 expression in samples treated with ML364 at the *IC*_*50*_ concentration.** A** and **B** Cell viability in D283 Med and USP-13 cells was assessed by using a range of ML364 concentrations. Viability was assessed at 24, 48, or 72 h after treatment. Both cell lines showed a dose- and time-dependent decrease in viability. Two-way ANOVA test. **(*p* = 0.0028) ****(*p* < 0.0001). **C** and **D** Lower USP2 level (23%) in D283 Med cells. No statistically significant differences were observed between groups. Note: CT = Control group

Treating D283 Med or USP-13 cells with a specific inhibitor of the USP2 protein, the compound ML364, at concentrations ranging from 5 to 150 µM for 24, 48, or 72 h. While an increased viability was observed at the lowest tested concentration (5 µM), ML364 consistently induced a dose- and time-dependent reduction in cell viability at concentrations ≥ 25 µM. (Fig. 5A and B). The CompuSyn software (ComboSyn Inc., Paramus, NJ, USA) was used to calculate the ML364 *IC*_*50*_ concentration for these cell lines. At 48 h, the *IC*_*50*_ of ML364 was 61 and 52 µM for D283 Med and USP-13 cells, respectively. Treatment with ML364 at the *IC*_*50*_ concentration reduced USP2 expression in D283 Med cells by 23%. (Fig. 5C and D).

### ML364 Inhibited Colony Formation in D283 Med and USP-13 Cells

**Fig. 6:**
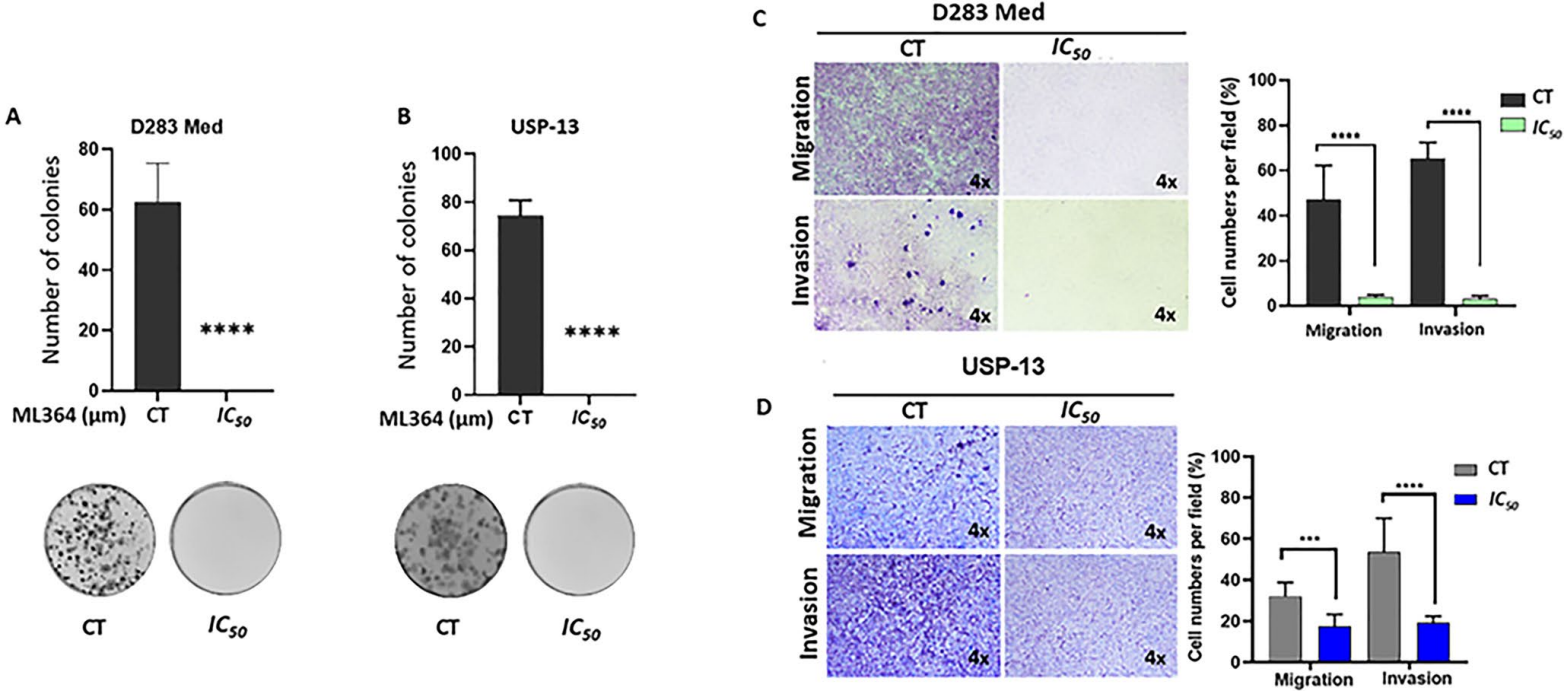
Clonogenic and transwell assays conducted in D283 Med and USP-13 cells treated with ML364.** A** and **B** Clonogenic assay showing that lost their ability to form colonies after treatment with ML364. Mann-Whitney test. ****(*p* < 0.0001). **C** and **D** Decreased migration and invasion capacity following treatment with ML364 at the *IC*_*50*_ concentration. Mann-Whitney test. ****(*p* < 0.0001). Note: CT = Control group

We plated and treated D283 Med or USP-13 cells with ML364 at the *IC*_*50*_ concentration and evaluated the ability of the cells to form colonies. Both cell lines showed totally reduced colony formation ability (Fig. 6A and B). ML364 inhibited the migration and invasion capacity of D283 Med and USP-13 cells. On the basis of the Transwell assay, the migration and invasion capacity of D283 Med cells decreased from 47.1% to 3.9% and from 65.5% to 3.2%, respectively, after treatment with ML364 at the *IC*_*50*_ concentration for 48 h. As for USP-13 cells, the migration and invasion capacity also decreased, from 32% to 17.5% and from 53.5% to 19.1%, respectively, after treatment with ML364 at the *IC*_*50*_ concentration for 48 h (Fig. 6C and D).

### USP2 Acts on Proteins Related to the Cell Cycle

**Fig. 7:**
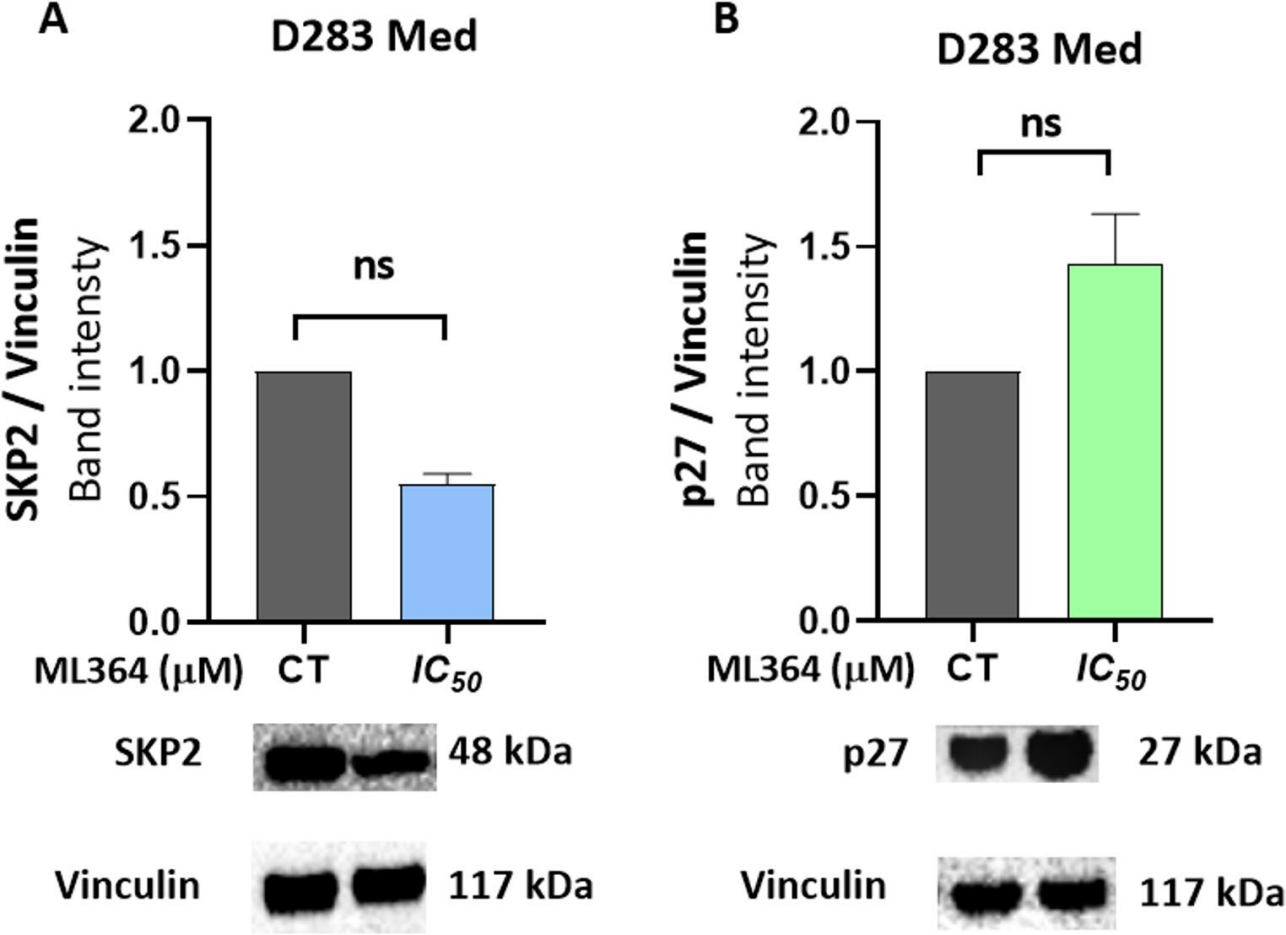
Effects of ML364 treatment on SKP2 and p27 protein expression in D283 Med cells.** A** Densitometric analysis and representative Western blot bands of SKP2 protein levels normalized to Vinculin after ML364 treatment at the *IC*_*50*_ concentration. **B** Densitometric analysis and representative Western blot bands of p27 protein levels normalized to Vinculin under the same experimental conditions. Data are expressed as mean ± SD (or SEM). Statistical analysis was performed using the Mann-Whitney test. No statistically significant differences were observed between groups. Note: CT = Control group

To analyze the role played by USP2 in proteins involved in cell cycle processes, we investigated whether SKP2 expression changed in D283 cells treated with ML364 at the *IC*_*50*_ concentration. Indeed, SKP2 expression decreased about 40% (Fig. 7A). Additionally, expression of the tumor suppressor p27 increased in the same proportion, as expected (Fig. 7B), despite not showing statistical significance.

## Discussion

Medulloblastoma is an aggressive malignant neoplasm with high mortality and morbidity rates, especially in patients diagnosed with SHH MB with *TP53* mutations, G3 MB with *MYCC* amplification, or G3 MB with metastasis. These MB molecular groups are categorized on a high-risk scale and have five-year overall survival rate of less than 50% [20–22]. Developing research to characterize new therapeutic targets for this neoplasm is essential.

During previous research, our group used RNA sequencing analysis (RNAseq database – GSE181293) and identified differentially expressed genes (DEGs) in G3 MB [6]. By employing in silico analyses using public databases of pediatric MBs (GSE85217) [3], our group identified the Ubiquitin Specific Peptidase 2 (*USP2*) gene as a biomarker of poor prognosis in MB. To date, the role played by *USP2* in G3 and G4 MBs has not been investigated. The USP2 protein is known to act in the dissociation of free or protein-bound ubiquitin, thereby allowing previously ubiquitylated proteins to escape proteasomal degradation and to perform their functions in controlling cell cycle, proliferation, and migration [23, 24]. In contrast, USP2 overexpression impairs the normal functioning of these molecular processes in the cellular microenvironment and culminates in dysregulated mitotic cycle, thus increasing tumor cell proliferation, migration, and invasion [25]. This occurs because some molecules that would normally be degraded by the proteasomal complex through the action of USP2 remain active in the cellular environment, leading to tumor initiation and progression [7, 15, 16]. USP2 exists in several tissues and is expressed in different cells and organs, such as the heart, liver, kidney, breast, brain, and skeletal muscle [26, 27]. However, *USP2* is overexpressed in metastatic lesions of triple-negative breast cancer and has been associated with chemotherapy resistance in prostate, lung, and colorectal cancers. Furthermore, *USP2* overexpression has been correlated with increased tumor cell proliferation, migration, and invasion in ovarian cancer and urothelial and hepatocellular carcinomas [13, 15, 16, 28, 29]. The results of the present study on *USP2* expression conducted in samples obtained from the FMRP/CIB cohort, as well as the results of the in silico analyses of the GSE85217 database show that *USP2* is overexpressed in G3 and G4 MBs and metastatic tumors, which is associated with worse prognosis [3, 5]. In this same database, higher *USP2* expression is associated with lower overall survival, both in the general MB molecular groups and G3/G4 MBs. Similar results have been described in triple-negative breast and colorectal cancers, in which *USP2* is overexpressed [7, 30, 31]. *USP2* can potentially discriminate between WNT/SHH MBs and G3/G4 MBs as evidenced by the ROC curves in our cohort and in the GSE85217 database. This suggests that *USP2* expression indicates specific groups with worse prognosis.

We observed that the treatment of G3/G4 MB cell lines with ML364, a selective USP2 inhibitor [32], at its *IC*_*50*_ concentration reduced USP2 expression by only 23% in D283 Med cells; however, no reduction was observed in the USP-13 cell line (data not shown). Nevertheless, because ML364 acts post-translationally, it competitively binds to target sites, which may explain this small decrease in its expression [33]. ML364 proved to reduce colony formation, migration, and invasion of D283 and USP-13 cells effectively, mainly in the case of the D283 Med cell line. *USP2* and USP2 expression levels are higher in D283 Med than in USP-13 cells, which may account for the different percentages of migrated and invaded areas between the two cell lines. High *USP2* expression has been associated with increased cell proliferation, migration, and invasion in triple-negative breast and bladder cancers [7, 34, 35]. In lung cancer, *USP2* overexpression has been described to promote migration and invasion of A549 cells, but the migratory and invasive capacity of tumor cells has been shown to decrease after they are treated with ML364 [25]. Other studies in choroidal melanoma have also shown that migration and invasion decrease after treatment with ML364 [36, 37]. Studies have demonstrated the role played by USP2 in proteins involved in the cell cycle: USP2 stabilizes these substrates and ensures that they function during cell division [38]. In lung cancer, USP2 has been reported to act on SKP2, a protein involved in many biological processes, particularly in the cell cycle. SKP2 participates in the degradation of tumor suppressors, specifically p27 and p21 [39]. Thus, we investigated p27 and SKP2 expression in D283 cells treated with ML364 at the *IC*_*50*_ concentration, to find that SKP2 decreases and p27 increases. These findings are consistent with previous reports describing the USP2/SKP2/p27 regulatory axis and suggest that USP2 inhibition may influence pathways associated with cell-cycle regulation. However, dedicated cell-cycle assays were not performed in the present study, and therefore direct effects of USP2 inhibition on cell-cycle progression cannot be concluded.

Together, our findings have allowed us to verify, for the first time, that *USP2* may contribute to the aggressiveness of tumor phenotypes observed in G3 and G4 MBs. In addition, we have observed that ML364 can be used to treat such MBs. Although USP2 is expressed in normal tissues, the antitumoral effects observed following ML364 treatment suggest that G3/G4 MBs may be dependent on USP2 activity. These findings support the potential use of USP2 inhibitors as a targeted therapeutic strategy, although further in vivo studies are required to assess safety and therapeutic selectivity. Indeed, this compound reduces the features that contribute to the aggressiveness of G3 and G4 MBs, which opens new possibilities for understanding the molecular mechanisms underlying these MBs and may guide the development of more effective therapeutic strategies to target the most aggressive G3/4 MBs.

## Supplementary Information

Below is the link to the electronic supplementary material.

Supplementary Material 1

## Data Availability

The data generated in the present study may be requested from the corresponding author.
