# Supplementary material for: Therapeutic Potential of Targeting USP2 in Pediatric Group 3 and 4 Medulloblastomas: Insights from In Silico, Ex Vivo, and In Vitro Studies
Source: Cerebellum. 2026 Jul 9;25(4):107. doi: 10.1007/s12311-026-02051-w (PMC13350141; doi:10.1007/s12311-026-02051-w)
Supplement: Supplementary file 1 — Supplementary Material 1 [file 12311_2026_2051_MOESM1_ESM.pdf]

Supplementary Table 1. *USP2* and *HPRT* primer sequences

Gene	Primer Sequence
USP2	Forward 5’CCGACAGATGTGGAGAAA3’
	Reverse 5’TTAGCAATGGGCAGTGAG3’
HPRT	Forward 5’TGGCGTCGTGATTAGTGATG3’
	Reverse 5’GCCTCCATCTCCTTCATC3’

Supplementary Table 2. Biological characteristics of the D283 Med and USP-13 Cell lines of MBs

Cell Line	Age	Gender	Molecular Subgroup	*TP53* Mutation	*MYC* Amplification	*OTX* Amplification	Reference
**D283 Med**	6-y	Male	Group 3/4	No	Yes	Yes	[1,2]
**USP-13**	3-y	Male	Group 3/4	No	No	Yes	[3]

1. Friedman HS, Burger PC, Bigner SH, Trojanowski JQ, Wikstrand CJ, Halperin EC, Bigner DD. Establishment and characterization of the human medulloblastoma cell line and transplantable xenograft D283 Med. J Neuropathol Exp Neurol. 1985 Nov;44(6):592-605. doi: 10.1097/00005072-198511000-00005.
2. Sengupta S, Weeraratne SD, Sun H, Phallen J, Rallapalli SK, Teider N, Kosaras B, Amani V, Pierre-Francois J, Tang Y, Nguyen B, Yu F, Schubert S, Balansay B, Mathios D, Lechpammer M, Archer TC, Tran P, Reimer RJ, Cook JM, Lim M, Jensen FE, Pomeroy SL, Cho YJ. α5-GABAA receptors negatively regulate MYC-amplified medulloblastoma growth. Acta Neuropathol. 2014 Apr;127(4):593-603. doi: 10.1007/s00401-013-1205-7.
3. Silva PB, Rodini CO, Kaid C, Nakahata AM, Pereira MC, Matushita H, Costa SS, Okamoto OK. Establishment of a novel human medulloblastoma cell line characterized by highly aggressive stem-like cells. Cytotechnology. 2016 Aug;68(4):1545-60. doi: 10.1007/s10616-015-9914-5.

Supplementary Table 3. List of antibodies used in the study.

Antibody	Code	Dilution	Specification	Company
**USP2**	MBS920 6307	1:2000 5% BSA in TBS-T	Anti-rabbit Policlonal	MyBioSource
**VINCULIN**	sc- 73614	1:1000 5% milk in TBS-T	Anti-mouse Monoclonal	Santa Cruz Biotechnology (Santa
**SKP2**	DEG5	1:1000 5% BSA in TBS-T	Anti-rabbit Policlonal	Cruz, California, USA) Cell Signalling
**p27**	Kip 1 D69C12	1:1000 5% BSA in TBS-T	Anti-rabbit Policlonal	Cell Signalling

Supplementary Table 4 – Clinical characteristics of the FMRP/CIB cohort

Clinical Variable	Number of patients (%)
**Gender**	
Female	21 (38.9%)
Male	33 (61.1%)
**Age at diagnosis**	
< 3 years	4 (7.4%)
> 3 years	50 (92.6%)
**Degree of surgical resection**	
Complete	36 (66.7%)
Incomplete	18 (33.3%)
**Metastasis**	
Absence	38 (70.4%)
Presence	16 (29.6%)
**Recurrence**	
Yes	15 (27.8%)
No	38 (70.4%)
NE	1 (1.8%)
**Event**	
Alive	31 (57.4%)
Recurrence/Death	23 (42.6%)
**Disease status**	
Alive	31 (57.4%)
Death (MB)	21 (38.9%)
Other Causes	2 (3.7%)

*NE: Not Evaluated
